# Conservation of Nonsense-Mediated mRNA Decay Complex Components Throughout Eukaryotic Evolution

**DOI:** 10.1038/s41598-017-16942-w

**Published:** 2017-11-30

**Authors:** Barry Causier, Zhen Li, Riet De Smet, James P. B. Lloyd, Yves Van de Peer, Brendan Davies

**Affiliations:** 1 Centre for Plant Sciences, Faculty of Biological Sciences, University of Leeds, Leeds, LS2 9JT UK; 2 Department of Plant Biotechnology and Bioinformatics, Ghent University, Technologiepark 927, B-9052 Gent, Belgium; 3 VIB Center for Plant Systems Biology, Technologiepark 927, B-9052 Gent, Belgium; 4 Department of Genetics, Genomics Research Institute, University of Pretoria, Private Bag X20, Pretoria, 0028 South Africa

## Abstract

Nonsense-mediated mRNA decay (NMD) is an essential eukaryotic process regulating transcript quality and abundance, and is involved in diverse processes including brain development and plant defenses. Although some of the NMD machinery is conserved between kingdoms, little is known about its evolution. Phosphorylation of the core NMD component UPF1 is critical for NMD and is regulated in mammals by the SURF complex (UPF1, SMG1 kinase, SMG8, SMG9 and eukaryotic release factors). However, since *SMG1* is reportedly missing from the genomes of fungi and the plant *Arabidopsis thaliana*, it remains unclear how UPF1 is activated outside the metazoa. We used comparative genomics to determine the conservation of the NMD pathway across eukaryotic evolution. We show that SURF components are present in all major eukaryotic lineages, including fungi, suggesting that in addition to UPF1 and SMG1, SMG8 and SMG9 also existed in the last eukaryotic common ancestor, 1.8 billion years ago. However, despite the ancient origins of the SURF complex, we also found that SURF factors have been independently lost across the Eukarya, pointing to genetic buffering within the essential NMD pathway. We infer an ancient role for SURF in regulating UPF1, and the intriguing possibility of undiscovered NMD regulatory pathways.

## Introduction

**Figure 1:**
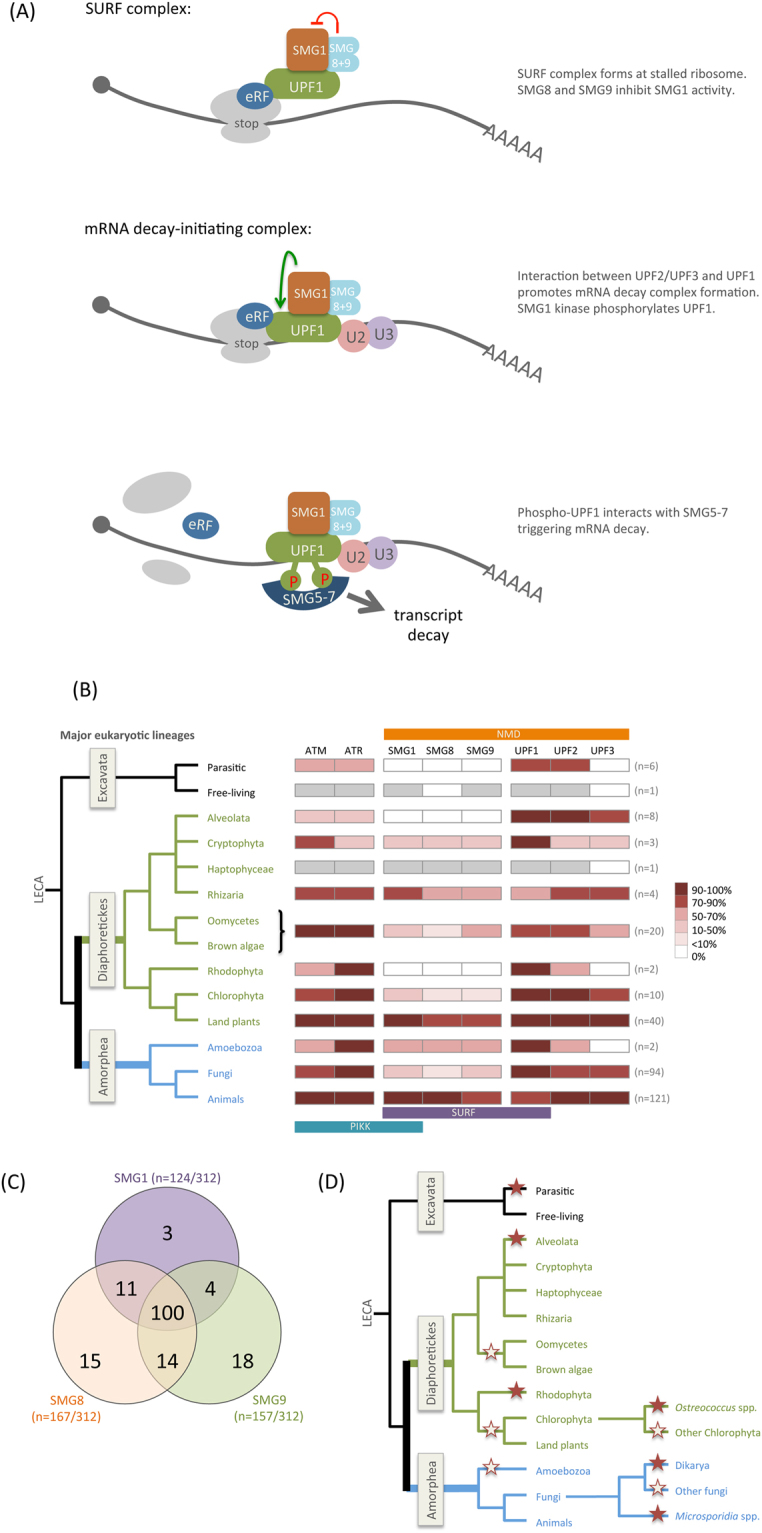
NMD factors and PIKKs have been retained and lost throughout eukaryote evolution. (**A**) Key steps in the NMD process. (1) The transient SURF complex (SMG1 + UPF1 + eRFs) forms at a ribosome stalled at a stop codon. In the SURF complex, SMG8 and SMG9 prevent UPF1 activation by inhibiting its phosphorylation by SMG1. SMG1, SMG8 and SMG9 are also important for recruiting UPF1 into the SURF complex. (2) Following interaction between UPF1 and the core NMD factors UPF2 and UPF3, the SURF complex is remodelled into a transcript decay-initiating complex. This disrupts the SMG8/SMG9-mediated inhibition of SMG1, allowing it to phosphorylate and activate UPF1, which interacts with transcript decay-inducing factors, including SMG5-7 (3). (**B**) A total of 312 eukaryote genomes were surveyed for the presence or absence of genes encoding core NMD factors, PIKKs and subunits of the SURF complex. Numbers to the right indicate the number of individual species within a particular eukaryotic group that were examined. The summarized phylogeny to the left shows the relationships between the major eukaryotic groups studied. The percentage of species within each group where genes encoding these factors were found is represented by heat maps, as shown to the right. Where only a single representative genome was available for a particular group, only presence (grey box) or absence (white box) is shown. (**C**) Venn diagram showing the number of eukaryote genomes from which genes encoding components of the SMG1C (SMG1, SMG8 and SMG9) were found to be absent. (**D**) The coding capacity for the entire SMG1C has been lost at the base of major eukaryote lineages (represented by filled stars on the phylogeny). Independent losses of the SMG1C was also found within clades (open stars on phylogeny).

Transcript decay is a key determinant of gene expression levels and the rate of change of expression^1,2^. Eukaryotic cells have evolved pathways to regulate transcript abundance, both to modulate expression and for quality control. Nonsense-mediated mRNA decay (NMD) is a quality control mechanism that eliminates aberrant mRNAs containing premature translation termination codons (PTCs). NMD also influences the abundance of up to 10% of non-aberrant transcripts in animals, fungi and plants^3–8^, suggesting that it is an important post-transcriptional regulator of gene expression. NMD is activated when translating ribosomes stall at termination codons in specific contexts^9–13^. In mammals, the first step in NMD is formation of a transient ‘SURF’ complex on the stalled ribosome. SURF is composed of SMG1, SMG8 and SMG9 (collectively known as SMG1C^14^), together with the core NMD factor UPF1 and eukaryotic release factors 1 and 3 (eRF1 and 3)^14,15^. Phosphorylation of UPF1 by the SMG1 phosphatidylinositol 3-kinase related kinase (PIKK), is central to NMD^16^. SMG8 and SMG9 act as a scaffold for the interaction between SMG1 and UPF1, but also negatively regulate SMG1 kinase activity, maintaining UPF1 in an unphosphorylated, inactive state within the SURF complex^14,17,18^. Interaction between UPF1 and the other core NMD factors, UPF2 and UPF3, promotes formation of an mRNA decay-initiating complex (Fig. 1A). UPF2 and UPF3 antagonise the inhibitory effects of SMG8 and SMG9, allowing SMG1 to phosphorylate and activate UPF1 (reviewed^11^). In common with other PIK-kinases, SMG1 phosphorylates Serine (S) or Threonine (T) residues within a S/TQ dipeptide motif^19^. NMD requires phosphorylation of the N- and C-termini of the UPF1 protein, which are enriched for S/TQ dipeptides, leading to recruitment of SMG5, SMG6 and SMG7 by phospho-UPF1 and transcript decay^16,20,21^ (Fig. 1A).

UPF1 and SMG1 are multifunctional proteins with cellular roles independent of NMD. In metazoans UPF1 is a component of the Staufen-mediated mRNA decay pathway^22^. In addition, UPF1 is an important regulator of genome integrity^23–27^. Similarly, SMG1 has been implicated in DNA damage responses and identified as a tumor suppressor linked to different cancers, stimulating interest in SMG1 as a novel target for drug therapies and a biomarker for cancer prognosis^11,28,29^. SMG1, and the related PIKKs ATM and ATR regulate genome integrity, through phosphorylation of both UPF1 and p53. However, an NMD compromised phenotype has only been observed for mutants or knockdowns of SMG1^24,30^, suggesting that SMG1 is the only PIKK that phosphorylates UPF1 to promote NMD, at least in metazoans.

Our understanding of NMD is less advanced outside of animal systems, but similarities and differences are emerging. For example, although UPF1 is phosphorylated in the model fungus *Saccharomyces cerevisiae* ^31,32^, the *SMG1* kinase gene is missing from its genome^33^. *SMG1* is also missing from the genome of the model plant *Arabidopsis thaliana*, although UPF1 is phosphorylated in plants at the N- and C-termini, which act redundantly in NMD^34,35^. The apparent lack of a kinase to activate UPF1 raises unresolved questions about the mechanism of NMD in these organisms.

Until recently it was thought that SMG1, and hence the SURF complex, was only found in animals^36,37^. However, we recently demonstrated that *A. thaliana* is atypical, since SMG1 is widespread in the plant kingdom, where it also functions in NMD^38^. The involvement of SMG1 in NMD in both animals and plants indicates that its NMD function likely predates the divergence of eukaryotes. However, loss or depletion of SMG1 in different branches of the eukaryote domain paints a more complex picture. Depletion of SMG1 is fatal in mammals^39^ and disrupts NMD in *C. elegans* and the moss plant *Physcomitrella patens* ^33,38^. In contrast, evolutionary losses of *SMG1* from *S. cerevisiae* and *A. thaliana*, or induced loss of *SMG1* in *Drosophila* and zebrafish are tolerated^40,41^.

Here we take a genomic approach to provide a framework to interpret the functional data from different species and to understand the origins, mechanism and evolution of NMD. By mapping the distribution of SURF complex components (UPF1, SMG1, SMG8 and SMG9), other core NMD factors (UPF2 and UPF3) and PIKKs (ATM and ATR) across a comprehensive selection of 312 genomes spanning eukaryotic evolution we provide evidence that all known SURF complex components were present in the last eukaryotic common ancestor (LECA). However, our discovery of multiple independent losses of the SURF complex during eukaryotic evolution indicates the existence of undiscovered NMD regulatory processes, which might also be of ancient origin. Our findings have implications for the fields of gene regulation and RNA processing.

## Results

By screening 312 eukaryotic genomes we determined the current distribution of the genes encoding the components of the NMD-promoting SURF complex (*SMG1, SMG8, SMG9, UPF1*), other core NMD factors (*UPF2, UPF3*) and other PIK kinases that target UPF1 (*ATM, ATR*). The complete dataset is presented in Tables S1–S3.

### Components of the NMD pathway were present in the earliest eukaryotes

A recent global phylogeny classified eukaryotes into three megagroups^42^: the Excavata, the Amorphea (which includes animals, fungi and Amoebozoa), and the Diaphoretickes (plants, Stramenopiles, Alveolata and Rhizaria) (see Fig. 1B). As an early branching sister group to the eukaryotes, the Excavata have been the focus of considerable study to understand eukaryotic origins^42^. Since NMD has been shown to be at least partially active in members of the Excavata group^43–45^, we searched the genomes of seven Excavata species for genes encoding NMD and SURF factors (Fig. 1B and Table S1). Although UPF1 is conserved in all seven species, components of the SMG1C complex (SMG1, SMG8 and SMG9) that regulates UPF1 activity for NMD in mammals are restricted to *Naegleria gruberi*, which has both SMG1 and SMG9. *N. gruberi* is a free-living organism, whereas the other Excavates used in this study are obligate parasites, which are often characterised by highly reduced genomes (reviewed^46^). To our knowledge, this is the first report of SMG1C components in these deep diverging lineages of eukaryotes. A previous study^43^ reported the identification of an *SMG1* homologue in *Giardia lamblia* (Genbank accession no. AY095369), but BLAST homology searches and protein domain analyses clearly show this to be more closely related to a different PIKK (mTOR) than to SMG1.

In animals UPF1 phosphorylation occurs when the core NMD factors UPF2 and UPF3 interact with the SURF complex to form an mRNA decay-initiating complex (Fig. 1A). UPF1 and UPF2 have previously been reported in yeast, animals, plants and the Excavata^11,44^ and we also find UPF1 and UPF2 in all major eukaryotic lineages (Fig. 1B and Table S1). UPF3, another core component of the NMD pathway, is poorly conserved, making identification of UPF3-encoding genes problematic^47^. *UPF3* was duplicated at the base of the vertebrates to yield two paralogs (*UPF3A* and *UPF3B*), which appear to act antagonistically in NMD^48^. Outside of the vertebrates, a single *UPF3* gene was found in each species apart from the Excavata (Table S1). Nevertheless, since *UPF3* is present in genomes from the Amorphea and Diaphoretickes (Fig. 1B), it seems likely that UPF3 also has an ancient origin.

Our data supports the notion that core components of the NMD pathway, including the auxiliary components of the SURF complex SMG8 and SMG9, emerged in the earliest eukaryotes. This view is reinforced by the finding that archaea genomes contain genes encoding UPF1-like DNA/RNA helicases, but not other core NMD components or PIKKs (Table S4).

### PIKK regulators of UPF1 activity are ancient protein kinases

The phosphorylation of UPF1 by PIKK kinases is crucial for its varied activities. While SMG1 is the only PIKK to activate UPF1 for both NMD, SMG1 and the related PIKKs ATM and ATR all phosphorylate UPF1 upon DNA damage^25,30,49^. We find *SMG1, ATM* and *ATR* genes in the genomes of species from both the Amorphea and the Diaphoretickes (Fig. 1B, Tables S1 and S3). In contrast to a previous report^50^, the *N. gruberi* genome contains genes encoding SMG1, ATM and ATR (Table S3). Other studies have identified *ATM* and/or *ATR* in the parasitic Excavate species *Trichomonas*, *Leishmania* and *Trypanosoma*, but not *Giardia* ^50–52^. Consistent with this, we found *ATM* and *ATR* genes in the genomes of *Trichomonas vaginalis*, *T. brucei* and *Leishmania major*, but not in *Giardia intestinalis, G. lamblia* or *Spironucleus salomonicida* (Table S3).

These findings indicate an ancient origin for PIK kinases, although we also observe rare, independent PIKK losses throughout eukaryote evolution. Of the eight Alveolata genomes examined, we failed to identify *ATM* or *ATR* in six. The Alveolata can be separated into three distinct groups: ciliates, dinoflagellates and apicomplexans^53^. Loss of *ATM* and *ATR* from both *Paramecium tetraurelia* and *Tetrahymena thermophila* suggests that these PIKKs were lost at the base of the ciliates (Table S3). No dinoflagellate genomes were examined here, but both *ATM* and *ATR* were found within the apicomplexans (*Plasmodium vivax* and *Toxoplasma gondii*), although *ATR* was missing from all 5 *Plasmodium* species examined, while *ATM* was only found in *P. vivax*. Together, this suggests multiple losses of these kinases during Alveolata evolution. *SMG1* is also missing from all Alveolata genomes examined (see below), which suggests that these largely parasitic eukaryotes can survive without UPF1-activating PIKKs. We were also unable to find *ATM* in the genomes of certain fungal groups, including the Glomeromycota and its sister group the Mucoromycota, suggesting loss of *ATM* from the common ancestor of these fungi. *ATM* is also missing from the majority of Microsporidia genomes examined (Table S3), suggesting that it has been lost independently throughout fungal evolution.

ATM, ATR and SMG1 are found throughout the Eukarya domain, yet despite their ancient origins our findings suggest that some eukaryotes can tolerate the loss of all UPF1-activating PIKKs, implying that there are alternative mechanisms to regulate UPF1 activity.

### SMG1 has been independently lost from diverse eukaryotic lineages

**Figure 2:**
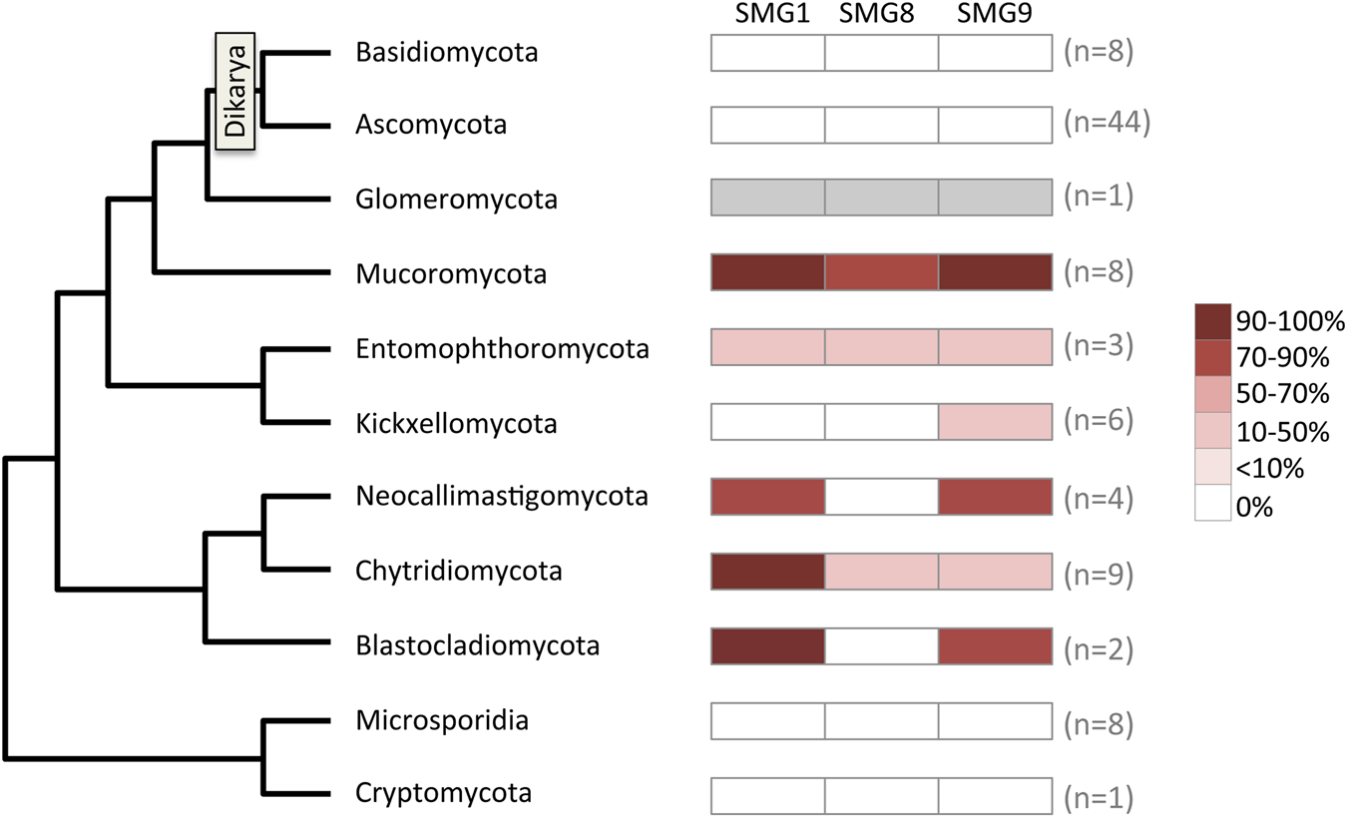
The fungal kingdom has retained SMG1. A total of 94 fungal genomes were surveyed for the presence or absence of genes encoding the individual members of the SMG1C (SMG1, SMG8 and SMG9). The summarized phylogeny to the left shows the relationships between fungal groups studied. The percentage of species within each fungal group where SMG1C-encoding genes were found is represented by the heat map, as shown to the right. Where only a single representative genome was available for a particular group, only presence (grey box) or absence (white box) is shown.

SMG1, the only kinase known to activate UPF1 for NMD, is essential for mammalian viability^39^, but its role in other species is less clear^40,41^. Phylogenetic profiling revealed that *SMG1* is missing from 124 of the 312 genomes surveyed (Fig. 1C and Table S1). Many of the observed *SMG1* losses are from fungi (*SMG1* is in just 24 of the 94 fungal genomes examined, Table S1). *SMG1* is absent from Ascomycota and Basidiomycota fungal genomes (collectively known as the Dikarya; Fig. 2). Phylogenetic analyses place the Glomeromycota (the arbuscular mycorrhizae) and Mucoromycota as sister groups to the Dikarya^54^ (see Fig. 2). Despite the close position of these fungal groups to the Dikarya, from which *SMG1* is absent, *SMG1* is retained in genomes of both Glomeromycota and Mucoromycota (Fig. 2 and Table S1). Furthermore, we also found *SMG1* in genomes of a number of basal fungal groups (Fig. 2). Therefore, in contrast to current opinion^37^, *SMG1* is present in the genomes of several fungal clades although multiple independent losses of *SMG1* have occurred throughout fungal evolution.

**Figure 3:**
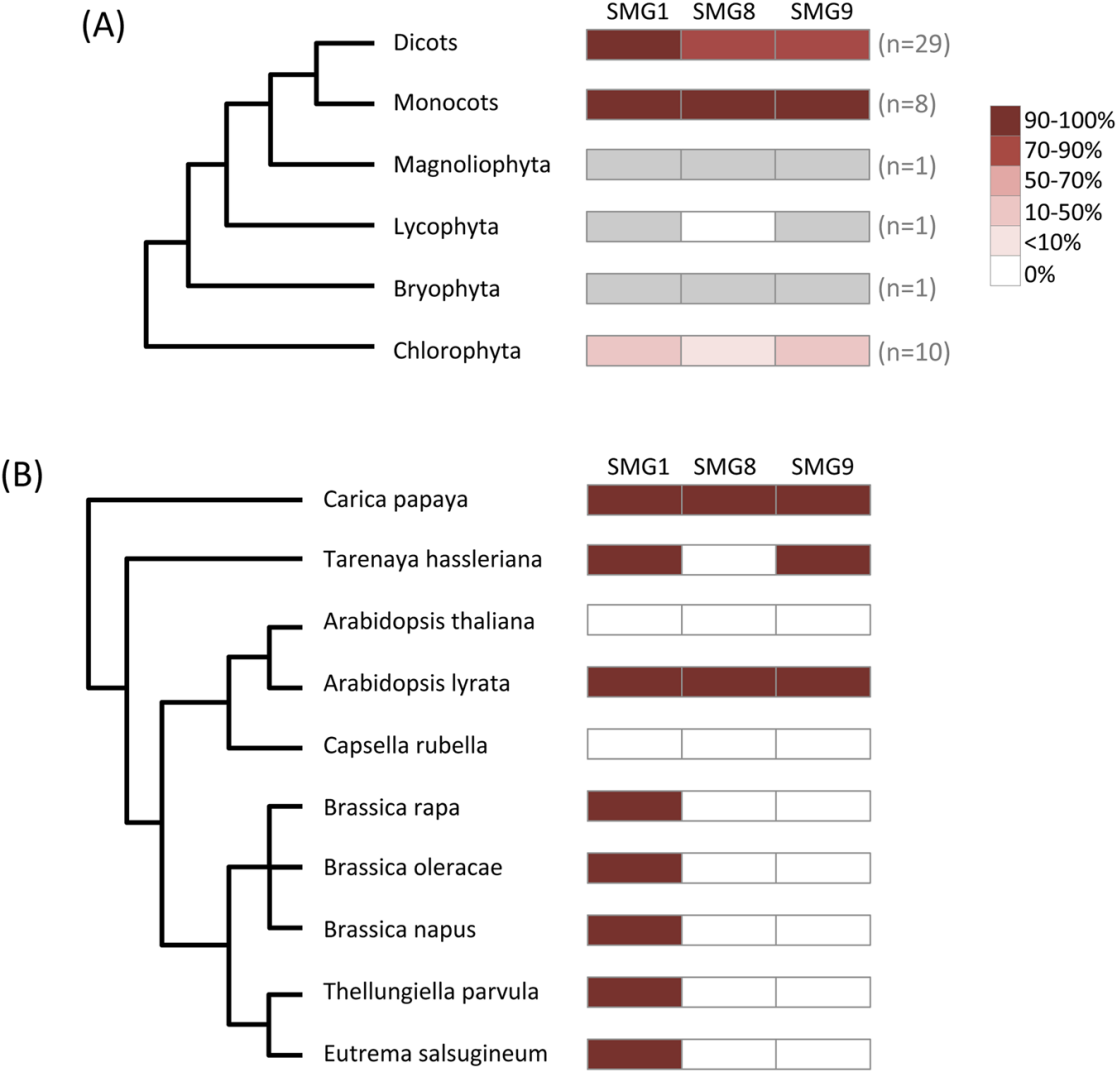
Plants show independent losses of SMG1. A total of 50 green plant genomes were surveyed for the presence or absence of genes encoding the individual members of the SMG1C (SMG1, SMG8 and SMG9). The phylogenies to the left show the relationships between the plants studied. (**A**) All major green plant groups have retained the gene encoding the PIK kinase SMG1. Loss of other components of the SMG1C vary between lineages. The percentage of species within each plant group where SMG1C-encoding genes were found is represented by by the heat map, as shown to the right. Where only a single representative genome was available for a particular group, only presence (grey box) or absence (white box) is shown. (**B**) Within the angiosperms (flowering plants), *SMG1* loss is restricted to the Brassicas, which includes the model plant *Arabidopsis thaliana*. Gene presence (red box) or absence (white box) is shown for each species.

The fungal and animal kingdoms both belong to the Amorphea megagroup of eukaryotes (Fig. 1B). Nevertheless, in contrast to the high frequency of loss of *SMG1* from fungal genomes, loss of *SMG1* from animal genomes is extremely rare (Fig. 1B). Of the 121 animal genomes examined, we failed to find *SMG1*-like genes in only three instances: *Mnemiopsis leidyi* (Eumetazoa), *Trichinella spiralis* (Nematoda) and *Megaselia scalaris* (Arthropoda) (Table S1). The Diaphoretickes megagroup of eukaryotes includes plants, Stramenopiles, Alveolata and Rhizaria (see Fig. 1B). *SMG1* is absent from 12 of the 19 Stramenopile genomes examined and all of the Alveolata genomes (Table S1). In contrast, *SMG1* is present in the vast majority of green plant genomes, including relatives of the earliest aquatic plants (the Chlorophyta), the earliest diverging land plants (e.g. the moss *Physcomitrella patens*), the earliest diverging vascular plants (e.g. the lycophyte *Selaginella moellendorffii*), and flowering plants (Fig. 3A and Table S1). Loss of *SMG1* was only seen in the Chlorophyta (60% of the examined genomes lacked *SMG1*) and a single group of land plants, the Brassicaceae, to which the model plant *Arabidopsis thaliana* belongs (Fig. 3B). As previously reported^38^, *SMG1* is missing from the genome of *A. thaliana* yet is present in the genome of its nearest relative *Arabidopsis lyrata*. Since these species diverged only 5–10 mya^55^ this represents a recent independent loss (Fig. 3B).

In summary, while *UPF1* has been maintained in almost all eukaryotic genomes, *SMG1* has been independently lost in many lineages. Despite its absence from the genome of the model species *S. cerevisiae* and most fungal groups, *SMG1* has been retained in at least four major fungal lineages. In contrast, losses of *SMG1* from animal and land plant genomes are rare.

### SMG1C components are co-eliminated

The SMG1C complex includes SMG1 and the factors that control it in mammalian NMD. Phylogenetic profiling studies suggest that functionally linked genes are often gained and lost together in evolution^56,57^. To examine whether components of the SMG1C have been retained or lost in concert across eukaryotic evolution, we surveyed their phylogenetic distribution. *SMG1*, *SMG8* and *SMG9* are missing from 124, 167 and 157 of the 312 genomes examined, respectively, with loss of all three genes being found in 100 cases (Fig. 1C). Underlying this is a small number of SMG1C losses at the base of major clades (Fig. 1D). For example, all three SMG1C genes are missing from the Ascomycota and Basidiomycota fungal genomes, suggesting that the entire SMG1C was lost from the common ancestor of the Dikarya. The full complement of SMG1C genes was also likely lost from the common ancestor of Microsporidia. Beyond the fungi, patterns of gene loss suggest that the entire SMG1C was also lost at the base of the Alveolata (Fig. 1D). In addition to the loss of SMG1C at the base of major clades, many independent losses of the entire SMG1C can be seen within different lineages. For example, within the Chlorophyta all SMG1C genes are missing from all species of *Ostreococcus* examined (Fig. 1D).

There are a limited number of examples of more recent co-elimination of genes encoding SMG1C components. For example, the plant *A. thaliana* split from its nearest relative *A. lyrata* just 5–10 mya^55^ yet *A. thaliana* has lost all of the SMG1C genes, whilst they have all been retained in *A. lyrata* (Fig. 3B and Table S1). Genome synteny reveals that the expected locations of *SMG1*, *SMG8* and *SMG9* map to distinct regions of the *A. thaliana* genome (Figure S1), showing that each gene was lost independently. Fragments of *SMG1* and *SMG9* are detectable in the *A. thaliana* genome (Figure S1), confirming these recent gene losses. Examination of patterns of SMG1C gene loss in land plants suggests a complex history within the Brassicaceae, which includes *Arabidopsis*. As mentioned above, SMG1C is missing from *A. thaliana* but is retained in *A. lyrata*. All three SMG1C components appear to be lost in *Capsella*, which is sister to *Arabidopsis* (Fig. 3B), with a fragment of *SMG1* remaining that is different to that found in the *A. thaliana* genome (Figure S1a). Therefore, SMG1C has been independently lost at least twice in the Brassicaceae. Loss of individual SMG1C components is commonplace amongst the Brassicas. The extraordinary propensity of Brassicaceae to independently lose genes encoding SMG1C components, which contrasts starkly with their retention in other plant groups, could be explained by an alternative mode of NMD regulation in this plant group.

**Figure 4:**
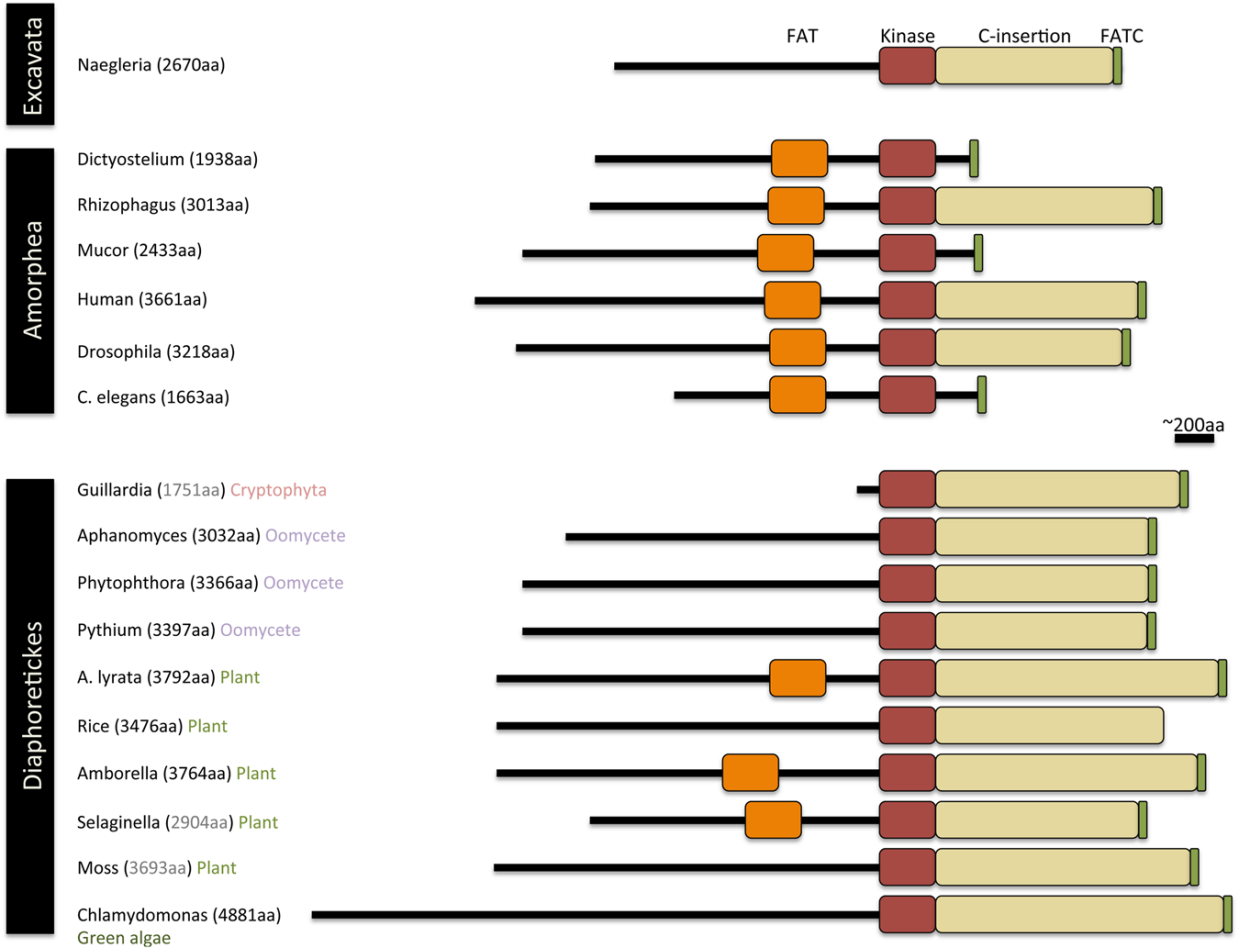
The domain structure of SMG1 proteins from the Amorphea and Diaphoretickes. The SMG1 kinase is characterised by three domains, the FAT domain (orange), the kinase domain (red) and the FATC, located at the C-terminus of the peptide (green). In addition, SMG1 contains a large C-insertion of approximately 1000 amino acids (yellow), situated between the kinase domain and the FATC. The structure of SMG1 is shown for the Excavata (*N. gruberi*), the Amorphea and the Diaphoretickes. Note that in the Diaphoretickes the C-insertion is consistently present, while in the Amorphea it is missing from SMG1 in several species.

Assembly of the SMG1C depends on a domain of SMG1 known as the C-insertion, which is not found in other PIKKs^58^. In mammals, the C-insertion is bound by SMG8 and SMG9 and recruits and regulates UPF1 activity and NMD. The retention of SMG1C throughout eukaryote evolution suggests that this regulatory mechanism is also conserved. However, the *C. elegans* SMG1 protein lacks the C-insertion and SMG8 is reportedly not required for nematode NMD^18,59^. To determine whether this may be the case in other eukaryotes, we examined SMG1 protein sequences for the presence of a C-insertion. We found that SMG1 proteins of all Diaphoretickes examined contain the C-insertion, as does the SMG1 protein from the Excavate *N. gruberi* (Fig. 4). In contrast, however, we identified multiple examples in different Amorphea lineages where the C-insertion is missing from SMG1 (Fig. 4). Although the C-insertion recruits SMG8 and SMG9, loss of this region from the SMG1 protein does not correlate with loss of SMG8 or SMG9. Taken together, the data suggest that the ancestral SMG1 protein contained the C-insertion and that it may have had the potential to recruit SMG8 and SMG9 into a regulatory SURF complex early in eukaryotic evolution. Furthermore, our findings suggest that in species where the C-insertion has been lost, alternative SURF-independent mechanisms of UPF1-regulation or alternative ways to recruit SMG8 and SMG9 could have evolved.

In summary, we find multiple independent instances of loss of the entire SMG1C, consistent with a mutually dependent role in NMD. The high frequency of losses of SMG1C components suggests that in many species, undiscovered SURF-independent mechanisms may exist to regulate the function of UPF1 in NMD, and that these too may be ancient in origin. Interestingly however, we did not find a single instance of complete loss of the SMG1C within the animal kingdom. It appears that different kingdoms, groups and species exhibit different capacities to tolerate the loss of SMG1C, possibly reflecting the efficiency of any alternative systems to regulate UPF1.

### Ancient loss of SMG1 correlates with co-loss of terminal UPF1 S/TQ dipeptide motifs

**Figure 5:**
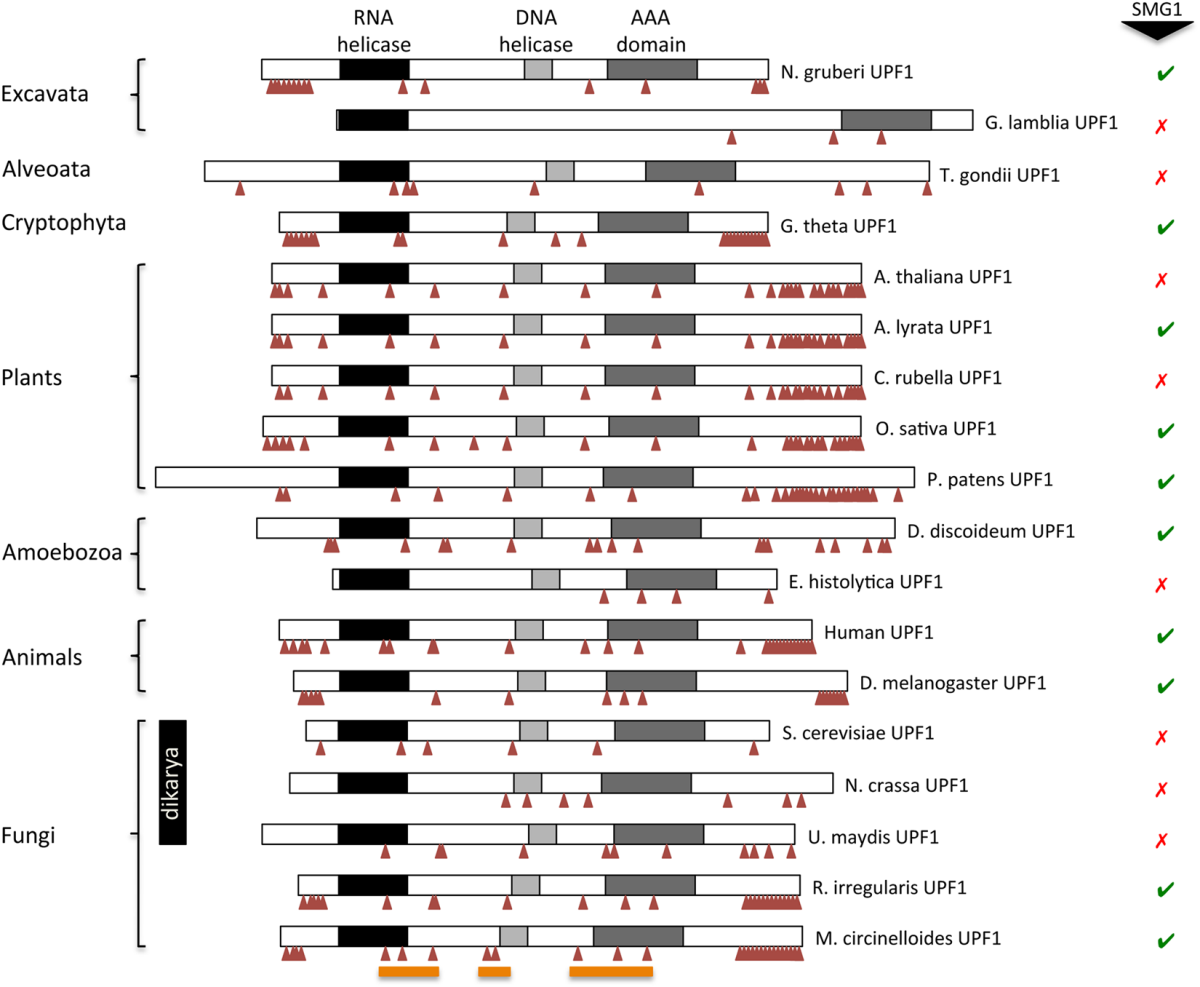
Mapping of S/TQ dipeptide motifs in UPF1 proteins from across the eukaryotes. The UPF1 protein contains a number of domains. Towards the N-terminus is a RNA helicase motif (black). Near the centre of the peptide is a DEAD-like DNA helicase domain (light grey). C-terminal to the DNA helicase is a AAA domain (dark grey). Red arrow heads indicate the approximate positions of SQ and TQ dipeptides within the UPF1 peptide sequence, which are thought to be the target sites for phosphorylation by the SMG1 kinase. Representatives from the major eukaryote groups are shown, as indicated to the left. The presence or absence of the *SMG1* gene in the genome of the relevant species is shown to the right. Orange bars at the bottom of the figure highlight S/TQ clusters maintained in species where loss of *SMG1* is predicted to have been an ancient event.

In mammalian NMD, SMG1 catalyses the phosphorylation of UPF1 at Serine or Threonine residues within clusters of S/TQ dipeptides at its N- and C-termini (Fig. 5). To understand how the multiple independent loss of *SMG1* could alter the way UPF1 is regulated, we compared the S/TQ dipeptide distribution in UPF1 in species where SMG1 was lost early or late in evolution. SMG1 was lost at the base of the fungal Dikarya group, over 580 mya, making this an ancient loss. Unlike human UPF1, Dikarya UPF1 proteins are relatively S/TQ poor at the N- and C-termini (Fig. 5 and Table S5). In contrast, UPF1 proteins from fungal lineages in which SMG1 has been retained contain extensive clusters of the S/TQ dipeptides at their N- and C-termini (Fig. 5). Similarly, the termini of UPF1 from *D. discoideum*, which retains SMG1, contain numerous S/TQ dipeptides, whereas loss of *SMG1* in *E. histolytica*, which split from *Dictyostelium* over 1 billion years ago, correlates with the UPF1 termini being relatively S/TQ-poor (Fig. 5). Loss of *SMG1* from the early branching Excavata also correlates with reduced S/TQ frequency in the associated UPF1 proteins.

In species where *SMG1* loss was more recent, extensive co-loss of S/TQ peptides in UPF1 would not be expected. *SMG1* was lost from the *Arabidopsis thaliana* genome just 5–10 mya and its UPF1 termini remain S/TQ rich (Fig. 5 and Table S5). Indeed, *A. thaliana* UPF1 shares an almost identical S/TQ distribution across the entire protein with that of *Arabidopsis lyrata*, which has retained *SMG1* (Fig. 5). Similarly, the UPF1 protein of *Capsella rubella*, which split from *Arabidopsis* approximately 14 mya^60^ is also S/TQ-rich, yet *SMG1* is missing (Fig. 5). The evolutionary analysis of S/TQ dipeptide distribution and SMG1 kinase conservation across eukaryote genomes leads to two predictions. Firstly, SMG1 is likely to phosphorylate N- and C-terminal clusters of S/TQ dipeptides in eukaryotic species beyond the animals. Secondly, the correlation between early evolutionary loss of SMG1 and a corresponding loss of terminal S/TQ clusters in UPF1 indicates that any alternative, or parallel UPF1 regulatory mechanism will not involve a PIK kinase acting in the same way as SMG1.

UPF1 is also phosphorylated by the PIKKs ATM and ATR, as a response to DNA damage. Like SMG1, ATM and ATR show specificity for S/TQ dipeptides. Since ATM and ATR do not appear to play a role in NMD, it is likely that the UPF1 phosphorylation events catalyzed by these PIK kinases differ from those catalyzed by SMG1. Thus, we might expect S/TQ dipeptides not targeted by SMG1 to be maintained in species that lack SMG1, but retain ATM/ATR. Consistent with this, the UPF1 N- and C-terminal S/TQs are preferentially lost in species lacking *SMG1*, while those distributed throughout the remainder of the protein are retained (Table S5). In particular, there are three clusters of S/TQ sites: one positioned at the C-terminal end of the RNA helicase domain, a second in and around the DNA helicase region, and a third in the proximity of the AAA domain of UPF1 in most species examined. These sites appear to be conserved from the earliest eukaryotes (Fig. 5 and Table S5).

## Discussion

The mammalian SURF complex regulates the activation of UPF1 by phosphorylation, which is a critical step in the essential NMD pathway. However, outside of mammals the role of the SURF complex in NMD is poorly understood. Until recently, SMG1 was thought to be an animal-specific NMD factor^36–38^, although even amongst animals its importance for NMD remains unresolved^39–41^. Recent data from plants revealed that not only does SMG1 exist beyond the animal kingdom it is also required for NMD^38^. Since SMG1 is found in all three of the eukaryote megagroups, it suggests that SMG1 was present in the LECA. Furthermore, the fact that SMG1 has a role in NMD in both animals and plants, which diverged over 1 billion years ago^61,62^, strongly supports the idea that one of the ancestral roles of SMG1 was regulation of NMD.

**Figure 6:**
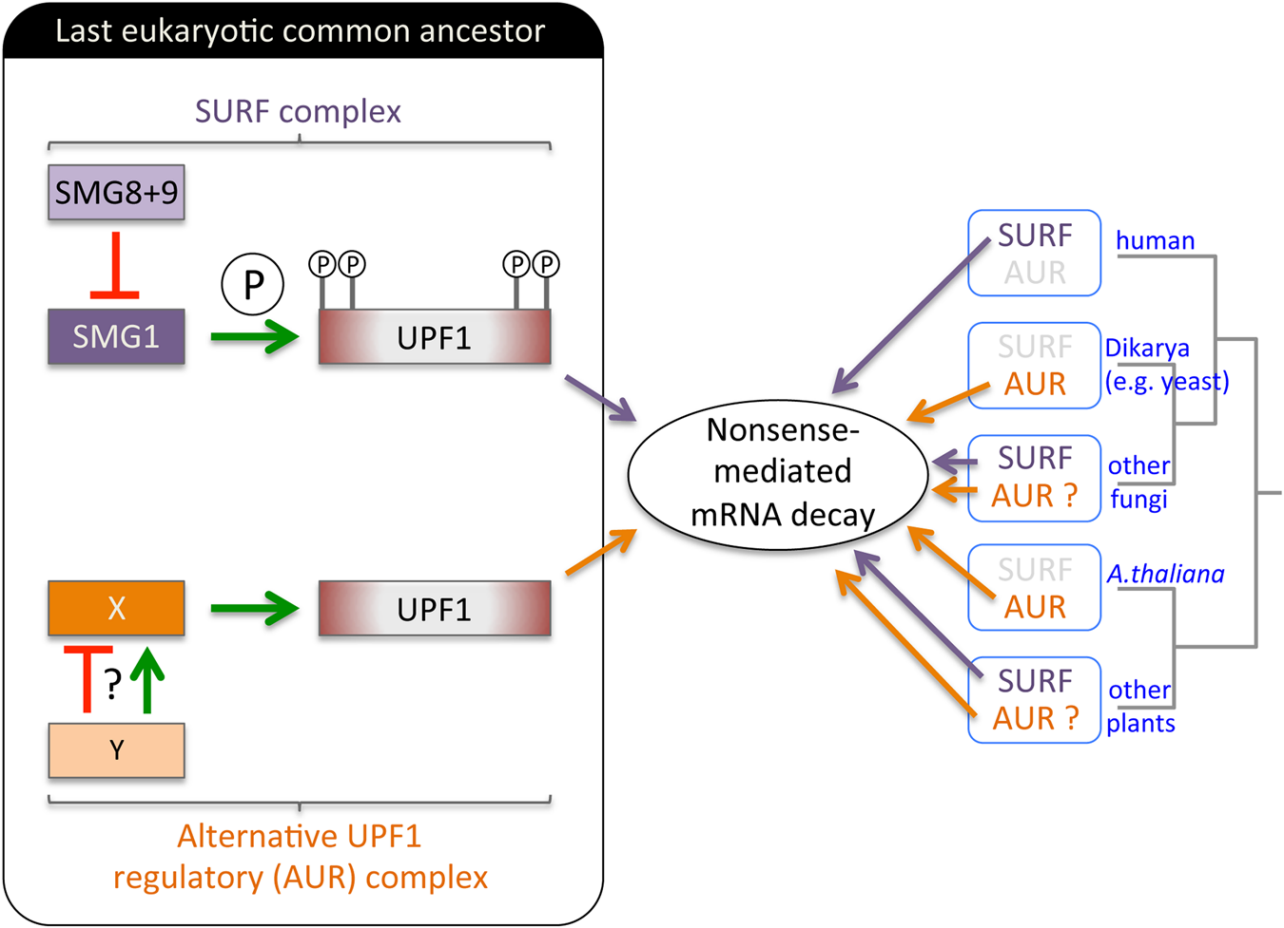
Model depicting the proposed evolution of protein complexes that regulate UPF1 for NMD. The model predicts the co-existence of the SURF complex and an alternative UPF1-regulatory (AUR) complex in the last eukaryotic common ancestor (left). Each complex was maintained throughout the evolution of all major eukaryote lineages. The presence of both complexes allows for genetic buffering in the regulation of UPF1 activation. It also permits independent evolutionary trajectories for the two complexes, since loss of one regulatory complex can be buffered by the presence of the other complex (right). Since the identity of AUR is unknown, it is also possible that only SURF was present in the LECA, and different alternative mechanisms to activate UPF1 could have evolved independently. Additionally, AUR may activate UPF1 through phosphorylation, since phosphorylation of UPF1 has been shown to be important in two species lacking SMG1, but other mechanisms to activate UPF1 may also exist.

Since protein complexes tend to form early in evolution and evolve as coherent units^63^, the presence of *SMG1* in the LECA prompted us to examine whether other components of the SURF complex were also present early in the evolution of eukaryotes. Others have previously shown that the SURF components eRF1 and eRF3 are conserved throughout eukaryotes^64,65^, so here we focused on UPF1, SMG1, SMG8 and SMG9. Phylogenetic profiling revealed that the SURF complex components are conserved in all major eukaryote groups (Fig. 1B), suggesting that the SURF complex was present in the LECA (Fig. 6). Despite this, the data presented here and elsewhere^11,38^, shows that SURF components have been independently lost multiple times throughout eukaryote evolution. It is well known that *SMG1* is absent from the *S. cerevisiae* genome and a previous analysis indicated that *SMG1* had been lost early in the fungal lineage, after divergence from the animals^38^. However, our analysis of 94 genomes spanning the fungal phylogeny reveals that major fungal groups, including the arbuscular mycorrhizae (Glomeromycota) and the pathogenic Mucoromycota, have retained *SMG1* (Fig. 2). Additionally, we show that *SMG1*, *SMG8* and *SMG9* were co-lost at the base of the Dikarya. In contrast to the fungi, loss of SMG1C components is rare in the animal kingdom, suggesting that while fungi can compensate for the loss of this NMD regulatory complex, this may not be the case for animals. Indeed, in mammals induced loss of *SMG1* is fatal^39^ and knockdown of *SMG1*, *SMG8* or *SMG9* impacts the health of the organism and is linked to pathological conditions in humans (reviewed^12^). Within the Diaphoretickes megagroup, we found multiple examples of *SMG1* loss from the Stramenopiles, the Alveolata, and from plants. Loss of *SMG1* from these groups, as with the fungi, correlates with loss of *SMG8* and *SMG9*. A common feature of genome evolution is that functionally linked genes are lost together^56,57^. Multiple independent, co-ordinate losses of SMG1C-encoding genes throughout eukaryote evolution (Fig. 1C), supports the hypothesis that SMG1, SMG8 and SMG9 have been functionally linked since the LECA.

Several important questions are raised by these findings. First, if the SURF complex was required for NMD in the LECA, how is NMD regulated in extant species lacking components of SURF proteins? For example, *S. cerevisiae* is used extensively as a model to study NMD, as is the plant *A. thaliana*. In both cases phosphorylation of UPF1 is required for normal NMD^32,34,35^. Phylogenetic reconstructions of ancestral gene content suggest that ancient eukaryotic genomes were rich in genes and protein domains^66,67^, and that descendant genomes have been shaped by pervasive differential gene loss^68^. However, evolution of the genome through reductive processes has, until recently, received scant attention^69^. It is now emerging that genome reduction is a major mode of evolution, allowing for adaptive genome streamlining that eliminates non-essential genes and other redundant genetic material^70^. In the case of the NMD pathway, preservation of NMD regulation in species such as *S. cerevisiae* and *A. thaliana* is most likely the consequence of genetic buffering^71^, where alternative functionally convergent pathways compensate for the loss of genes encoding the SURF complex. This would suggest that where SMG1 is missing, an alternative kinase, which may be an unrelated kinase, phosphorylates UPF1 for NMD^38^. The alternative kinase may have even existed in the LECA, activating NMD alongside SMG1 (Fig. 6). Throughout evolution either kinase may have been independently retained or lost, leading to the picture we see today where loss of SMG1 is tolerated due to genetic buffering (e.g. *S. cerevisiae* and *A. thaliana*), or is fatal (mammals), presumably because no fully redundant alternative kinase gene is present in these genomes (Fig. 6). The level of recurrent gene loss (recurring patterns of lineage specific gene loss) and the impact this has on genome evolution is not yet fully appreciated^69^. The lineage specific loss of genes such as *SMG1*, *SMG8* and *SMG9* across the Eukarya may add to the growing body of evidence for this evolutionary process. Second, where SMG1 is absent, which kinase(s) activate UPF1 for NMD? Phosphorylation of UPF1 by the PIKKs SMG1, ATM and ATR is stimulated in response to DNA damage. ATM and ATR are conserved in all eukaryotic groups (Fig. 1B and Table S3) and were likely present in the LECA, but in organisms lacking SMG1 there is no evidence that either ATM or ATR activate UPF1 for NMD^24,30,72–74^. In support of this we note that ancient loss of SMG1 is correlated with the co-loss of S/TQ dipeptide motifs at the N- and C-terminal ends of the UPF1 protein in these species (Fig. 5 and Table S5), suggesting that in these cases regulation of UPF1 activity occurs independently of the S/TQ motif. Recent mapping of the phosphorylation sites of *S. cerevisiae* UPF1 identified Tyrosine residues important for NMD, which are conserved across eukaryotes^32^, including one site conserved in the Excavate *N. gruberi*. It will be interesting to learn the identity of this new Tyrosine kinase, to determine whether it is evolutionarily conserved, and to discover whether it has a role in NMD across the eukaryotes.

In mammals, interaction between the SURF components SMG8-SMG9 and SMG1 requires the large C-insertion region of SMG1. The C-insertion, bound by SMG8 and SMG9, fulfills two roles: it acts as a platform for the recruitment of UPF1 into the SURF complex, and blocks UPF1 access to the active site of SMG1^18^, regulating UPF1 activity and therefore NMD. Here we show that the ancestral SMG1 protein contained the C-insertion, suggesting that SMG1 was able to recruit SMG8 and SMG9 into a regulatory SURF complex early in eukaryote evolution. Consistent with this, the SMG1 protein of the Excavate *N. gruberi* contains the C-insertion, as do all SMG1 proteins examined from the Diaphoretickes (Fig. 4). In contrast, there are multiple examples within Amorphea lineages where the C-insertion has been lost (Fig. 4). Deletion of the SMG1 C-insertion leads to hyper-activation of the kinase, indicating that the C-insertion allows fine-tuning of SMG1 activity^18^. It remains to be seen whether SMG1 lacking the C-insertion can recruit SMG8 and SMG9 to stabilise the UPF1-SMG1 interaction and regulate UPF1 phosphorylation. The finding that *C. elegans* SMG8 is not required for NMD^59^ suggests that this may not be the case and that protein complexes unrelated to SURF may regulate UPF1 activity in such species. It is tempting to speculate that a SURF-independent pathway of UPF1 and NMD regulation might have co-existed with SMG1 in the LECA and might still be present in some modern eukaryotes (Fig. 6).

We show that the components of the SURF complex, which regulates a key step in mammalian NMD, are widely conserved throughout eukaryotes, indicating an ancient origin. Although SMG1 is an effector of NMD in both animals and plants, it is unknown whether regulation of SMG1 activity is also conserved or how UPF1 is modified in species lacking SMG1. Our data provides a framework for future work that will establish how NMD is regulated amongst the different branches of the Eukarya.

## Materials and Methods

### Phylogenetic profiling

We downloaded proteins of 70 vertebrate genomes from Ensembl (release 77), 52 fungal genomes from Ensembl Fungi (release 24), 55 metazoan genomes from Ensembl Metazoa (release 24), and 37 protista genomes from Ensembl Protists (release 24). For green plants and algae, we used the 51 species available in PLAZA 3.0^75^ and pico PLAZA^76^. The proteins for the basal fungal lineages (i.e. in total 42 species from Cryptomycota, Microsporidia, Neocallimastigomycota, Chytridiomycota, Blastocladiomycota, Kickxellomycotina, Entomophthoromycotina, Mucoromycotina, and Glomeromycota) were retrieved from MycoCosm^77^.

We used BLASTP (2.2.30)^78^ to search for proteins from each of the species above that show sequence similarity to isoforms of nine human genes: SMG1 (Ensembl accession no. ENSP00000402515), SMG8 (Ensembl accession no. ENSP00000438748), SMG9 (Ensembl accession no. ENSP00000270066), UPF1 (Ensembl accession no. ENSP00000470142), UPF2 (Ensembl accession no. ENSP00000348708), UPF3A (Ensembl accession no. ENSP00000364448), UPF3B (Ensembl accession no. ENSP00000276201), ATM (Ensembl accession no. ENSP00000388058), and ATR (Ensembl accession no. ENSP00000343741). As an E-value cut-off for this BLAST-search 1 × 10^−3^ was used. For the best hits in each species a BLAST search against the human genome (E-value < 1) was performed to ensure that the hit corresponds to the query-gene as reciprocal best hit (RBH). Based on the outcome of this reciprocal blast analysis we constructed a phylogenetic profile matrix (Tables S1–S3). In this matrix, rows represent the target genomes and the columns the query-genes, whereas the values represent the bit scores of the RBH. Because gene length leads to a bias of higher bit scores for longer alignments, we normalized bit scores by the bit score of the query-genes themselves, i.e. the bit score corresponding to a BLASTP-search for a query-gene to itself. The normalized scores, which range from zero to one [0,1], stand for the similarities of RBH to the query genes with values closer to one indicating higher similarity^79^. In case only a one-directional hit was found we included the best hit gene in the human genome that corresponds to the hit. We performed the same analysis with eight genes in *Arabidopsis lyrata* by using SMG1 (Phytozome 12 accession no. AL3G35680), SMG8 (Phytozome 12 accession no. AL4G11880), SMG9 (Phytozome 12 accession no. AL6G16810), UPF1 (Phytozome 12 accession no. AL8G01130), UPF2 (Phytozome 12 accession no. AL4G25670), UPF3 (Phytozome 12 accession no. AL1G35280), ATM (Phytozome 12 accession no. AL5G17000), and ATR (Phytozome 12 accession no. AL7G37010) as query-genes (Tables S1–S3).

Additional BLASTP searches were performed at https://blast.ncbi.nlm.nih.gov/Blast.cgi for NMD-related genes in the plants Selaginella, *Brassica napus*, *Eutrema* and *Tarenaya*, the Rhodophyte *Galdieria*, the Oomycete *Aphanomyces* and the Excavates *T. vaginalis*, *S. salmonicida* and *G. intestinalis*. NMD genes were also identified in the Excavate *N. gruberi* using BLAST at http://genome.jgi-psf.org/pages/blast-query.jsf?db = Naegr1, and in plant *B. oleracae* using BLAST at http://brassicadb.org/brad/blastPage.php (Table S1).

### Genome synteny analysis

Synteny between the available plant genome sequences was detected essentially as previously described^80^. We used the Plant Genome Duplication Database (PGDD; http://chibba.agtec.uga.edu/duplication/index/home)^81–83^ to search for collinear genomic fragments between species based on *Arabidopsis lyrata* loci: *SMG1* (PGDD accession no. 16047708), *SMG8* (PGDD accession no. 16039886) and *SMG9* (PGDD accession no. 16037990). Fragments of *SMG1* and *SMG9* in the *Arabidopsis thaliana* genome, and *SMG1* in the *Capsella rubella* genome were detected by taking genomic sequence between flanking genes (based on synteny; see Figure S1) and using BLASTX against *A. lyrata* (https://blast.ncbi.nlm.nih.gov/Blast.cgi) to identify any sequences with homology to the NMD factors.

### Protein domain analysis

The conserved protein domains present in UPF1 sequences from the species listed in Table S5 were identified using NCBI Conserved Domain Search (https://www.ncbi.nlm.nih.gov/Structure/cdd/wrpsb.cgi?). Similarly, the positions of the SMG1 protein domains, and the presence/absence of the C-insertion were identified using the same program for the species shown in Fig. 4.

### Idenitification of UPF1 S/TQ dipeptides

S/TQ sites were mapped for the UPF1 peptide sequences from the species listed in Table S5, using NetworKIN (http://networkin.info)^84^.

All data generated or analysed during this study are included in this published article (and its Supplementary Information files).

## Electronic supplementary material

Supplementary Information with Figure S1 and Table S4

Table S1

Table S2

Table S3

Table S5
