# Supplementary material for: Conservation of Nonsense-Mediated mRNA Decay Complex Components Throughout Eukaryotic Evolution
Source: Sci Rep. 2017 Nov 30;7:16692. doi: 10.1038/s41598-017-16942-w (PMC5709506; doi:10.1038/s41598-017-16942-w)
Supplement: Supplementary file 1 — Supplementary Information with Figure S1 and Table S4 [file 41598_2017_16942_MOESM1_ESM.doc]

**Supplemental information**

**Conservation of Nonsense-Mediated mRNA Decay Complex Components Throughout Eukaryotic Evolution**

Barry Causier, Zhen Li, Riet De Smet, James P. B. Lloyd, Yves Van de Peer, Brendan Davies

Table S1, S2, S3 and S5 are Excel files included as separate files

Figure S1 and Table S4 are included in this document

**
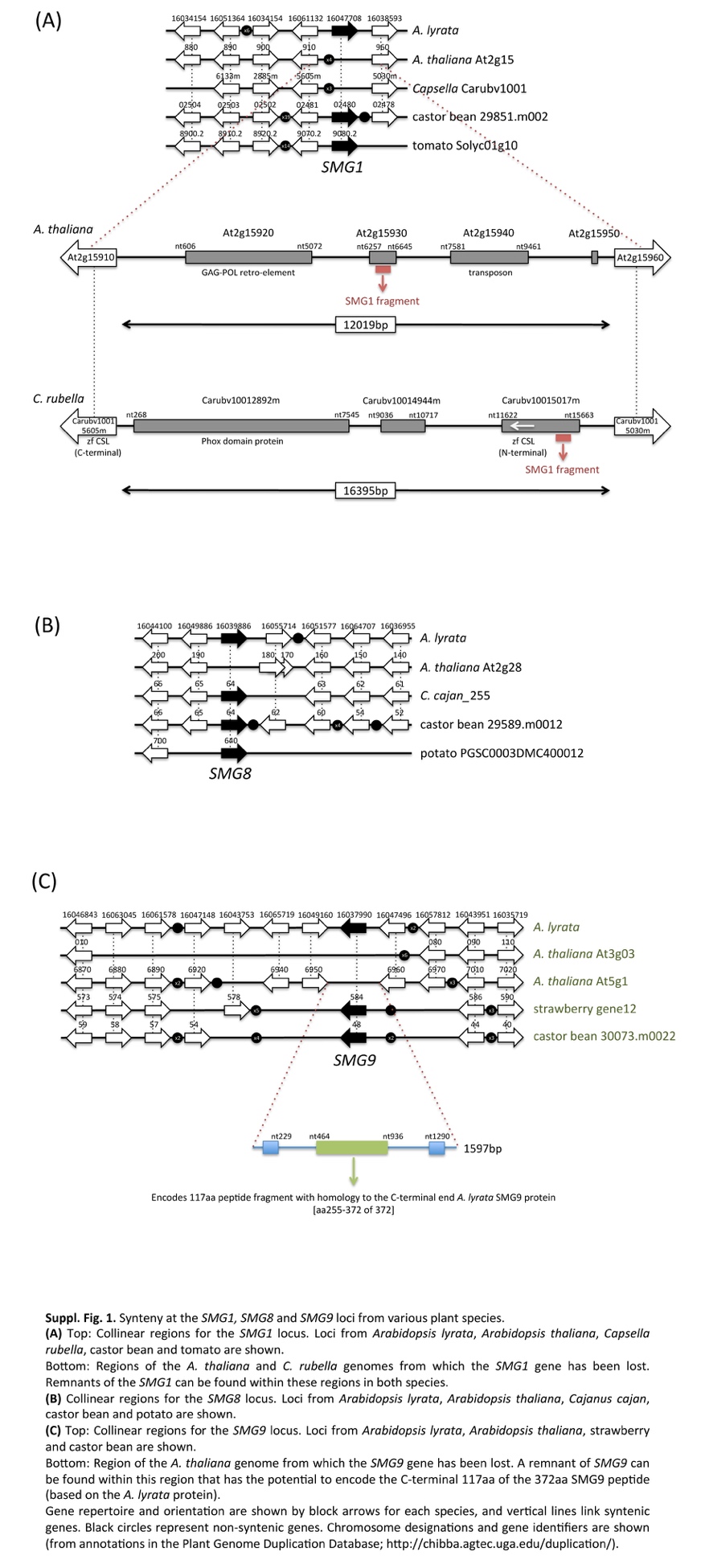
Figure S1**

**
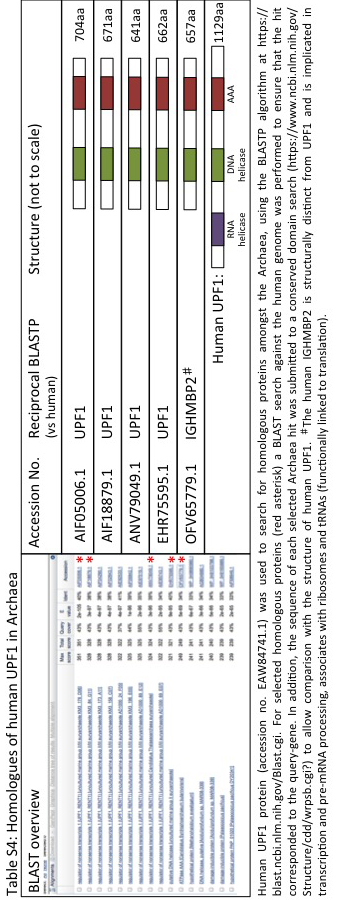
 Table S4**
